# Fusobacterium in the microbiome: from health to disease across the oral–gut axis and beyond

**DOI:** 10.1038/s41522-025-00838-z

**Published:** 2025-10-14

**Authors:** Zhengrui Li, Ji’an Liu, Jing Li, Zhaokai Zhou, Xufeng Huang, Divya Gopinath, Peng Luo, Qi Wang, Dan Shan

**Affiliations:** 1 Department of Oral and Maxillofacial & Head and Neck Oncology, Shanghai Ninth People’s Hospital, Shanghai Jiao Tong University School of Medicine, Shanghai, China; 2 College of Stomatology, Shanghai Jiao Tong University, Shanghai, China; 3 National Center for Stomatology, Shanghai, China; 4 National Clinical Research Center for Oral Diseases, Shanghai, China; 5 Shanghai Key Laboratory of Stomatology, Shanghai, China; 6 Shanghai Research Institute of Stomatology, Shanghai, China; 7 Shanghai Center of Head and Neck Oncology Clinical and Translational Science, Shanghai, China; 8 Research Unit of Oral and Maxillofacial Regenerative Medicine, Chinese Academy of Medical Sciences, Shanghai, China; 9 Shanghai Stomatological Hospital & School of Stomatology, Fudan University, Shanghai, China; 10 Department of Urology, The First Affiliated Hospital of Zhengzhou University, Zhengzhou, China; 11 Department of Urology, The Second Xiangya Hospital of Central South University, Changsha, China; 12 Faculty of Dentistry, University of Debrecen, Debrecen, Hungary; 13 Department of Data Visualization, Faculty of Informatics, University of Debrecen, Debrecen, Hungary; 14 Basic Medical and Dental Sciences Department, College of Dentistry, Ajman University, Ajman, UAE; 15 Centre of Medical and Bio-allied Health Sciences Research, Ajman University, Ajman, UAE; 16 Department of Oncology, Zhujiang Hospital, Southern Medical University, Guangzhou, China; 17 Department of Oncology, Ruijin Hospital, Shanghai Jiao Tong University School of Medicine, Shanghai, China; 18 Faculty of Health and Medicine, Lancaster University, Lancaster, UK

**Keywords:** Health care, Microbiology, Applied microbiology, Clinical microbiology, Microbial communities, Microbial genetics, Pathogens

## Abstract

*Fusobacterium* functions as both commensal and pathogen, linking the oral–gut axis to diverse diseases, including cancer. Evidence shows it modulates microbial balance, promotes dysbiosis, and contributes to carcinogenesis by driving inflammation, proliferation, invasion, and immune evasion. This review integrates ecological, molecular, and clinical insights, highlighting its roles in oral and systemic disease and discussing therapeutic potential, underscoring *Fusobacterium*’s dualistic nature and implications for microbiome-targeted interventions.

## Introduction

*Fusobacterium* occupies a distinctive niche in microbial pathogenesis, balancing between benign commensalism and opportunistic pathogenicity. This dual identity grants it a disproportionate influence within the human microbiome, shaping both local and systemic disease processes, from chronic infections to malignancies^1,2^. Compared with other oral microbes, *Fusobacterium* exhibits unique ecological and functional characteristics, notably its capacity to tolerate and persist under both aerobic and anaerobic conditions and its role as a “bridging organism” that links early and late microbial colonizers^3,4^. As commensal members of the human and animal microbiota, *Fusobacterium* species primarily inhabit mucosal surfaces, with the oral cavity and colon serving as major reservoirs. Among anaerobic Gram-negative bacteria, *Fusobacterium* stands out for its prevalence, intricate host interactions, and involvement in a wide spectrum of diseases. Its clinical importance is further underscored by associations with conditions ranging from periodontal diseases to colorectal cancer, implicating diverse and complex pathogenic mechanisms that merit in-depth investigation^5–7^.

Historically, research focused on its role in oral health, where *Fusobacterium* was recognized as a key architect of dental biofilms, contributing to periodontal disease and shaping oral microbial ecology^8^. Over the past few decades, however, interest has expanded markedly due to its strong association with colorectal cancer. The recognition of *Fusobacterium* as a potential oncogenic microbe represents a pivotal shift in understanding its role beyond the oral cavity. Initial studies documented its enrichment in colorectal tumors and its ability to promote tumor progression via immune modulation and chronic inflammation. Evidence now suggests that *Fusobacterium* could traverse anatomical boundaries, contributing to the pathogenesis of pancreatic, esophageal, and gastric cancers^9^. These findings raise the possibility that its presence may not be merely correlative but potentially causal in oncogenesis.

Mechanistic studies have identified key molecular pathways linking *Fusobacterium* to tumor biology. A landmark discovery was the binding of the *Fusobacterium* adhesin FadA to E-cadherin, a critical epithelial adhesion protein^10–13^. This interaction activates β-catenin signaling, driving cell proliferation, survival, and metastatic potential^12,14,15^. Such signaling perturbations not only promote tumor growth but also enhance invasiveness, positioning *Fusobacterium* as an active facilitator of malignant transformation.

Equally notable is *Fusobacterium’s* remarkable adaptability to diverse environments, ranging from oxygen-rich to strictly anaerobic niches, and its capacity to shift from a harmless commensal to a pathogen under dysbiotic or immunocompromised conditions^16–18^. This adaptability, coupled with its frequent association with aberrant tissue proliferation in multiple cancers, has earned it the designation of an “oncobacterium”^19,20^. Its dual nature presents both a challenge and an opportunity: understanding when and how *Fusobacterium* transitions from symbiont to pathogen may yield critical insights into microbe–host dynamics in health and disease.

In this review, we synthesize current knowledge on *Fusobacterium’s* ecological functions, its pathogenic mechanisms in infectious diseases and cancer, and its potential as a therapeutic target. By integrating ecological, molecular, and clinical perspectives, we aim to illuminate the multifaceted roles of Fusobacterium and outline avenues for innovative research and intervention strategies.

## Characteristics and significance

### Overview of *Fusobacterium*

*Fusobacterium* is an anaerobic, Gram-negative bacterial genus that exhibits diverse morphologies, ranging from spherical to rod-shaped forms, and is predominantly found in the human oral cavity and gastrointestinal tract^16,21–23^. Unlike most oral bacteria, *Fusobacterium* possesses a remarkable ability to maintain anaerobic metabolism under microaerophilic conditions, enabling it to persist in varied microbial communities. While typically commensal, certain species, most notably *Fusobacterium nucleatum* (*F. nucleatum*) and *Fusobacterium necrophorum* (*F. necrophorum*), are well-recognized pathogens capable of causing systemic infections across various age groups. Their abundance, measured in colony-forming units (CFU) from oral and stool samples, varies over the human lifespan, suggesting dynamic shifts in colonization patterns^24,25^. Distinct phenotypic traits, such as hemolytic activity and specific metabolic profiles observed in vitro, further aid in differentiating species and strains within the genus^26,27^.

Importantly, *Fusobacterium* exhibits a context-dependent duality: functioning as a benign commensal under normal physiological conditions but transitioning to a pathogen in dysbiotic or immunocompromised states. This switch underscores its relevance in both maintaining mucosal homeostasis and contributing to diverse disease processes when microbial balance is disrupted.

### Ecological interactions and diversity

*Fusobacterium* occupies a central ecological position in the oral and gastrointestinal microbiomes, acting as a bridging organism that facilitates co-aggregation between early colonizers (e.g., *Streptococcus* *spp.*) and late-stage anaerobes (e.g., *Porphyromonas* *spp.*). This bridging role promotes the hierarchical assembly and structural stability of biofilms. The process is mediated by specific molecular interactions, including FadA adhesins that bind both host tissues and neighboring bacteria, and virulence factors such as Fap2 that enhance co-aggregation and biofilm integrity.

Taxonomically, the genus comprises at least nine phylogenetic lineages and over 30 distinct species, as revealed by genomic sequencing^28^, marking a shift from earlier classifications based solely on metabolic and fermentative traits^29^. Ecologically, *Fusobacterium* shares close relationships with genera such as *Prevotella* and *Porphyromonas*, occupying overlapping niches and contributing to host cell invasion^2,4^. Its capacity to persist alongside oxygen-sensitive microbes even in oxygen-rich environments highlights its importance in maintaining microbial homeostasis under fluctuating oxygen conditions^8,16^.

Beyond its ecological contributions, *Fusobacterium* is implicated in a wide range of systemic conditions, particularly those affecting the oral and gastrointestinal tracts^30–33^. Its metabolic versatility, fermenting carbohydrates and proteins to produce short-chain fatty acids such as butyrate and acetate, affects host–microbe interactions in both healthy and dysbiotic states. Virulence factors, including leukotoxins and lipopolysaccharides (LPS), further enable its transition from commensal to pathogen, particularly in contexts of tissue damage or immune suppression^8,34^.

Within the genus, *Fusobacterium nucleatum subsp. animalis* has emerged as a clinically significant subspecies, especially in gastrointestinal diseases such as colorectal cancer and inflammatory disorders^28,35,36^. Recent studies indicate that this subspecies may play a prominent role in systemic infections and immune modulation. For example, it can bind to Siglec-7 receptors on natural killer (NK) cells, thereby suppressing NK cell-mediated cytotoxicity against cancer cells^37^. In addition, tandem mass spectrometry has been applied to detect *F. nucleatum* subspecies in the saliva from pre-colorectal cancer patients, underscoring the potential of subspecies-level detection as a biomarker in cancer-associated microbial communities^38^. Collectively, these findings position *F. nucleatum subsp. animalis* as a key focus for future research aimed at elucidating its ecological functions and pathogenic potential.

## Role of *Fusobacterium* species in oral diseases

*Fusobacterium* species are pivotal contributors to the pathogenesis of oral diseases, with their diverse virulence factors and complex interactions within microbial communities driving processes ranging from plaque formation and gingivitis to invasive infections. Among these, *F. nucleatum*, a prominent bacterium in human dental plaque, plays a crucial role in plaque formation and is implicated in conditions such as gingivitis^39,40^. Recent research highlights its ability to influence microbial community structure through adhesive interactions, forming microbial aggregates critical for plaque stability and pathogenicity. This capacity stems from its remarkable ability to adhere to a broad spectrum of Gram-positive and Gram-negative microorganisms within plaque, including *Porphyromonas gingivalis (P. gingivalis)*. Strongly linked to periodontitis, *F. nucleatum* is also frequently associated with invasive infections affecting the head and neck, thorax, lungs, liver, and abdomen. Its adhesive capabilities facilitate interactions with viruses and bacteria, promoting viral attachment to host tissue cells, thereby enhancing invasion and modulating host immune responses^41–44^.

In addition to *F. nucleatum*, *F. necrophorum* and other *Fusobacterium* species play significant roles in oral diseases. For example, *F. necrophorum* is frequently associated with severe forms of periodontal disease, particularly in immunocompromised patients^45–48^. These species contribute to pathogenic biofilm formation, and both *F. nucleatum* and *F. necrophorum* are implicated in the development of gingivitis, periodontitis, and more invasive oral infections.

### *Fusobacterium* in oral squamous cell carcinoma

Recent studies have identified an enrichment of *Fusobacterium* in oral squamous cell carcinoma (OSCC) tissues, as shown in Table 1^41,49–52^. The proportion of anaerobic bacteria is higher in OSCC tumors, whereas aerobic bacteria predominate in normal controls. These differences are primarily determined through sequencing methods, which, despite their high-throughput advantages, may be affected by technical biases such as DNA extraction efficiency or sequencing depth. This microbial shift highlights anaerobic bacteria as a potential reservoir on the OSCC surface, possibly contributing to cellular dysregulation. The distribution of *Fusobacterium* within OSCC tissues may differ greatly from that in healthy oral mucosa^53^. Notably, while several studies confirm the bacterium’s enrichment in OSCC, others report an absence of detectable *Fusobacterium* in cancer samples^53^ (Table 1).

**Table 1:** Associations and molecular mechanisms of *Fusobacterium* in various cancer types

Cancer Type	Association/Mechanism	Molecular Action
**Oral Cancer**	Enrichment in OSCC tissues	Increased presence in cancer tissues compared to healthy tissues.^41,51,52^
	Altered biofilm composition	A higher ratio of anaerobic-to-aerobic bacteria in OSCC biofilms^53^.
	Adherence and invasion	*F. nucleatum* adheres to and invades human gingival epithelial cells^58^.
	Specific bacterial components	Invasion requires specific bacterial components^2,4^.
	Lectin-like interactions	Involvement of lectin-like interactions in adhesion to OSCC cells^142,195^.
	Co-aggregation	*F. nucleatum* acts as a bridge for multispecies biofilm formation and facilitates colonization of *P. gingivalis* and *C. albicans*^165,166^.
**Colorectal Cancer**	Epigenetic changes	CpG island methylation, microsatellite instability, BRAF and TP53 mutations^100,133,134^.
	Cell proliferation	Activation of WNT/β-catenin signaling and TLR4 signaling through MYD88, increasing miR-21 expression^145^.
	Inflammation	Promotion of ROS generation and pro-inflammatory cytokines, activation of NF-κB pathway^73^.
	Immune modulation	Recruitment of MDSCs, inhibition of T cell proliferation, interaction with TIGIT receptor^142,150^.
	Migration and invasion	Induction of MMP-1 and MMP-9 production, downregulation of E-cadherin, upregulation of N-cadherin^163^.
**Esophageal Cancer**	Chemokine activation	Activation of CCL20 and inflammatory responses in tumor microenvironment^109^.
	m6A methylation	Increased METTL3-mediated m6A methylation promoting metastasis^110^.
	NF-κB pathway	Activation through NOD1/RIPK2 pathway, promoting tumor progression^111^.
**Gastric Cancer**	Microbiome alteration	Enrichment of *Fusobacterium, Lactobacillus*, and *Veillonella*, potential diagnostic biomarkers^112^.
	Gal-GalNAc antigen expression	High expression facilitates *Fusobacterium* enrichment, influencing disease progression^106^.
**Pancreatic Cancer**	Prognostic biomarker	Association with worse prognosis, colonization within PDAC tumors^9^.

This clinical association has prompted numerous in vitro studies exploring mechanisms by which *Fusobacterium* may mediate oral cancer progression^50,54–57^. *F. nucleatum* exhibits strong adherence to and invasion of human gingival epithelial cells (HGEC)^58^ (Table 1). It has also been shown to activate the NF-κB pathway, disrupt epithelial adhesion, and promote epithelial-mesenchymal transition (EMT), key steps in tumor progression and metastasis. Invasion appears to depend on specific bacterial components, as spontaneous mutants lacking invasive capabilities fail to penetrate HGEC^59,60^. Lectins, carbohydrate-binding proteins involved in cell–cell interactions, such as Galectin-9, are implicated in *F. nucleatum*-mediated adhesion and immune modulation in OSCC^61^ (Table 1).

Emerging evidence suggests that *Fusobacterium* does not act alone. Co-pathogens such as *P. gingivalis* and *Candida albicans (C. albicans)* have been observed to co-aggregate with *Fusobacterium* in biofilm communities. This co-localization enhances microbial colonization, sustains chronic inflammation, and may further promote oncogenic signaling in epithelial cells. Specifically, *P. gingivalis* could augment EMT via ZEB1-mediated signaling, while *C. albicans* may contribute to local acetaldehyde production, a known carcinogen. Furthermore, several studies link *Fusobacterium* presence to patient survival outcomes and clinicopathological parameters in oral cancer^62–64^. Notably, the detection of *Fusobacterium* correlates with clinicopathological parameters, including tumor size and stage^65^. This relationship suggests that *Fusobacterium* may influence disease progression and clinical prognosis, underscoring its importance in cancer biology.

*Fusobacterium*’s role also extends to interactions with human papillomavirus (HPV), particularly in head and neck cancers, suggesting that it can modulate immune responses and foster an inflammatory microenvironment that may exacerbate HPV-mediated carcinogenesis^66^. However, it is important to note that the interaction between *Fusobacterium* and HPV may vary among oral cancer subtypes. Some studies suggest mutual exclusivity between the two in certain head and neck cancers, indicating a complex interplay in cancer progression^67^

Multiple molecular pathways are activated by *Fusobacterium* in OSCC cells. Notably, the NF-κB pathway is markedly upregulated in its presence, promoting cancer cell survival, proliferation, and invasiveness^68^. Additionally, *Fusobacterium* can disrupt E-cadherin-mediated cell adhesion, facilitating EMT and metastasis^69,70^. These molecular interactions underscore the bacterium’s significant role in tumor progression and metastasis^71^.

A critical aspect of *Fusobacterium* infection is its induction of cytokine secretion. Studies have demonstrated that it stimulates the production of pro-inflammatory cytokines such as IL-6, IL-8, and TNF-α in oral cancer cells, contributing to a pro-tumorigenic environment^72^. This cytokine release can promote tumor growth, invasion, and resistance to apoptosis, highlighting the potential for targeting these inflammatory pathways as a therapeutic strategy.

### *Fusobacterium* as a periodontal pathogen

Toxic metabolites produced by *Fusobacterium* species can contribute to periodontal pathology by inhibiting or killing periodontal cells, such as fibroblasts^73–76^ (Table 1). Furthermore, *Fusobacterium*’s ability to evade immune responses through sulfide production and to modulate local inflammation underscores its role in chronic periodontal diseases^77^. For instance, sulfides generated by *F. nucleatum* may help the bacterium evade host immune defenses^78^. Butyric acid, along with propionic acid and ammonium ions produced by *F. nucleatum*, inhibits the proliferation of human gingival fibroblasts, further impairing periodontal tissue health^79^. *Fusobacterium* can also penetrate the gingival epithelium and is frequently found in plaque associated with periodontal disease, underscoring its prevalence and pathogenic potential^80^.

Beyond toxic metabolite production, *Fusobacterium* employs various molecular strategies to sustain infection within periodontal tissues^81–83^. Adhesins such as FadA facilitate bacterial adherence to and invasion of host epithelial cells, enabling deeper tissue colonization^13,84^. Furthermore, *Fusobacterium* modulates host immune responses by secreting enzymes and factors that degrade host tissues and immune components, thereby promoting bacterial survival and persistence. These toxins, while not directly cytotoxic, significantly impair fibroblast proliferation, delaying wound healing and contributing to chronic inflammation^85–87^. This interplay between bacterial virulence factors and host immune modulation highlights *Fusobacterium*’s pivotal role in the pathogenesis of periodontal disease, including gingivitis^88^.

## Role of *Fusobacterium* in gut cancers and diseases

The genus *Fusobacterium* exhibits a multifaceted role in gastrointestinal pathologies, spanning neoplastic and non-neoplastic diseases. Accumulating evidence implicates *Fusobacterium*, particularly *F. nucleatum*, not only in colorectal carcinogenesis but also in other gastrointestinal malignancies and inflammatory conditions, underscoring its systemic pathogenic potential.

### Impact of *Fusobacterium* on colorectal cancer

The *Fusobacterium* detected in colorectal cancer tissues is genetically identical to strains from the oral cavity, suggesting microbial translocation via hematogenous or enteric routes^89,90^. Whole-genome sequencing studies support this oral-gut axis, positioning *Fusobacterium* as a mediator of distal carcinogenesis. Clinically, meta-analyses demonstrate significantly higher *Fusobacterium* DNA detection rates in colorectal tumor tissues compared to adjacent healthy tissues or controls, with a less pronounced but still significant enrichment observed in precancerous polyps, particularly those with high-grade dysplasia^91^. Fecal microbiome analyses reveal elevated *F. nucleatum* abundance in colorectal cancer patients, with pooled odds ratios indicating stronger associations in colorectal cancer than in healthy controls or polyp-bearing individuals^23,92,93^. Beyond colorectal cancer, *F. nucleatum* has been detected in pancreatic, esophageal, and gastric cancers, suggesting broad oncogenic relevance^94,95^.

CpG islands are regions of DNA rich in cytosine and guanine dinucleotides, often located near gene promoters. In cancer, hypermethylation of these regions, frequently catalyzed by DNA methyltransferases (DNMTs), can silence tumor suppressor genes, contributing to tumorigenesis. The CpG Island Methylator Phenotype (CIMP) and Microsatellite Instability (MSI) are hallmark epigenetic and genetic alterations, respectively, in colorectal and gastric cancers, both of which have been linked to *Fusobacterium* presence. Specifically, studies demonstrate that *Fusobacterium* infection enhances DNMT-mediated CpG island methylation, which silences tumor suppressor genes such as MLH1 and CDKN2A, thereby driving cancer progression. Moreover, *Fusobacterium* triggers MSI through inflammatory responses that impair DNA mismatch repair proteins, further promoting genetic mutations. Mechanistic studies also show that *F. nucleatum* interacts with host cells via its FadA adhesin protein and E-cadherin, activating the β-catenin signaling pathway to promote tumor cell proliferation and invasion^13,96^. Additionally, *F. nucleatum* can inhibit the function of NK cells, diminishing immune surveillance and creating favorable conditions for tumor metastasis^97,98^.

Moreover, multiple studies associate intratumoral *Fusobacterium* with poorer survival outcomes in colorectal cancer^99,100^. *Fusobacterium* presence correlates with adverse clinicopathological features, such as larger tumor size, increased depth of invasion, poor differentiation, lymph node or distant metastasis, and advanced tumor stages^7,101–105^.

In summary, genomic analyses of the colorectal cancer microbiome consistently show significant enrichment of *Fusobacterium* species, particularly strains closely related to *F. nucleatum, F. mortiferum, and F. necrophorum*. These species have been identified in various clinical settings and are increasingly recognized for their potential role in human health and disease. This enrichment is corroborated by molecular analyses, such as fluorescence in situ hybridization (FISH), quantitative PCR, and sequencing-based methods, that identify *Fusobacterium* DNA within primary tumor tissues and colorectal metastases, highlighting its potential involvement in tumor progression and metastasis.

### Association of *Fusobacterium* with other gastrointestinal cancers

Recent studies reveal that beyond its established role in colorectal cancer, *Fusobacterium* is intricately linked to a spectrum of other gastrointestinal tumors, notably showing high prevalence in esophageal, gastric, and pancreatic cancers (Table 1). Pan-cancer analyses demonstrate an increased abundance of *Fusobacterium* in both primary tumors and adjacent normal tissues across gastrointestinal cancers, contrasting with non-gastrointestinal malignancies. Specifically, a significant prevalence of *Fusobacterium* species in gastric cancer patients suggests a potential contributory role in the pathogenesis of this cancer^106^. The enrichment of *F. nucleatum* in esophageal cancer has been correlated with poor prognosis^95,107,108^; however, direct mechanistic evidence remains limited.

CCL20 (C-C motif chemokine ligand 20) is a chemokine critically involved in inflammatory responses by recruiting immune cells, especially dendritic cells and Th17 cells, into inflamed tissues and tumor microenvironments. This association may be driven by *F. nucleatum*’s ability to activate chemokines such as CCL20, intensifying inflammatory responses within the tumor microenvironment^109^ (Table 1). Such an inflammatory milieu fosters a favorable environment for tumor progression.

Additionally, METTL3 (Methyltransferase-like 3) is an enzyme involved in m6A methylation, which plays a significant role in regulating RNA stability and splicing. Enhanced METTL3 activity has been associated with increased tumor metastasis and progression. Specifically, *F. nucleatum* infection has been shown to enhance METTL3-mediated m6A methylation, promoting metastasis in esophageal squamous cell carcinoma (ESCC)^110^ (Table 1).

Furthermore, NOD1 (nucleotide-binding oligomerization domain-containing protein 1) is an intracellular pattern-recognition receptor (PRR) that senses bacterial components, activating downstream signaling pathways involving the adaptor protein RIPK2 (receptor-interacting serine/threonine-protein kinase 2). *F. nucleatum* further invades ESCC cells, activating the NF-κB pathway via the NOD1/RIPK2 signaling axis, thereby facilitating tumor progression^111^ (Table 1). Collectively, these findings suggest that *F. nucleatum* contributes to cancer advancement through diverse mechanisms, including chemokine activation, epigenetic modifications, and inflammatory signaling.

In gastric cancer, the enrichment of bacterial taxa including *Fusobacterium, Lactobacillus, and Veillonella* underscores the significance of microbiome composition alterations in tumorigenesis^112^ (Table 1). Notably, *F. nucleatum* and other *Fusobacterium* species have emerged as potential diagnostic biomarkers for gastric cancer^113,114^. High expression of the Gal-GalNAc antigen, a carbohydrate structure serving as a binding target for specific bacterial adhesins, in gastric and esophageal cancers may facilitate increased adherence and enrichment of *F. nucleatum* within the tumor microenvironment, thereby influencing disease progression^115^.

In pancreatic cancer, while *Fusobacterium* spp. have been detected in pancreatic tumor tissues (Table 1), other studies have not consistently confirmed its involvement^116^. However, the presence of *Fusobacterium* within pancreatic ductal adenocarcinoma (PDAC) tumors correlates with a poorer prognosis in PDAC patients^117^. This observation suggests that *Fusobacterium* may serve as a prognostic biomarker for PDAC, indicating potentially variable roles of *Fusobacterium* across gastrointestinal cancers, possibly mediated by distinct mechanisms^95^.

In summary, the extensive presence of *Fusobacterium* across gastrointestinal tumors, particularly its dominance in the colorectal cancer microenvironment, highlights the complex and multifaceted relationship between *F. nucleatum* and gastrointestinal cancers. However, the evidence linking *Fusobacterium* to non-colorectal gastrointestinal cancers, such as pancreatic and esophageal cancer, remains largely correlative, and further mechanistic studies are needed to clarify its causal role in these contexts.

### Association of *Fusobacterium* with other gastrointestinal diseases

The relationship between *Fusobacterium* and inflammatory bowel disease (IBD) is particularly noteworthy within the broader context of digestive system disorders. IBD, which includes ulcerative colitis (UC) and Crohn’s disease (CD), is characterized by chronic intestinal inflammation. Recent research has increasingly focused on the genus *Fusobacterium*, especially *F. nucleatum*, due to its association with IBD^118,119^. Studies have demonstrated that both the frequency and abundance of *F. nucleatum* in the intestines of IBD patients are significantly elevated compared with healthy individuals, correlating closely with disease severity^120^.

*F. nucleatum* appears to drive UC progression by promoting a shift toward pro-inflammatory M1-type macrophages. Mechanistically, this occurs in part through activation of the AKT2 signaling pathway, an intracellular serine/threonine kinase pathway that regulates diverse cellular processes including proliferation, survival, metabolism, and inflammation. Targeting *F. nucleatum* or its AKT2-mediated inflammatory signaling could therefore represent a promising therapeutic approach for UC^121^. Furthermore, *F. nucleatum* exacerbates intestinal inflammation, epithelial barrier dysfunction, dysbiosis, and metabolic disruption, thereby worsening UC through its capacity to adhere to and invade host epithelial cells^122^.

The association between *Fusobacterium* and IBD underscores its broader role in digestive disease pathogenesis. Its high invasiveness and positive correlation with disease activity suggest that *F. nucleatum* may promote inflammation and disease progression by modulating host immune responses and destabilizing the intestinal microenvironment. Recent studies have further implicated *Fusobacterium* in functional gastrointestinal disorders, such as irritable bowel syndrome (IBS) and functional dyspepsia^123–125^. In these conditions, *F. nucleatum* can exacerbate symptoms by altering intestinal microbiota composition, disrupting the mucosal barrier, increasing intestinal permeability, and facilitating the release of inflammatory mediators^126^. Interactions between *Fusobacterium* and abnormalities in the enteric nervous system may also contribute to visceral hypersensitivity and motility disorders in IBS^127^. These findings suggest that rebalancing the intestinal microbiota, particularly by controlling *Fusobacterium* overgrowth, could offer a potential strategy for managing functional gastrointestinal diseases^128,129^.

Although direct studies on the association between *Fusobacterium* and liver diseases are limited, emerging research has begun to explore its potential impact^130^. In liver diseases, especially non-alcoholic fatty liver disease (NAFLD) and liver fibrosis, recent findings indicate a close link with gut microbiota dysbiosis^131^. *Fusobacterium* may exacerbate liver damage by directly influencing hepatocyte function and structure through its virulence factors^132^.

*Fusobacterium* species are also implicated in various gastrointestinal infections^31^. They have been isolated from intra-abdominal abscesses, liver abscesses, and colonic lesions. *F. nucleatum*, in particular, has been linked to colorectal cancer, where it promotes tumor growth and metastasis through interactions with host immune cells and the tumor-associated infectious microenvironment^31^.

In summary, the genus *Fusobacterium* demonstrates complex and multifaceted associations with a variety of non-tumoral digestive diseases. These connections enhance our understanding of the pathogenesis of these conditions and suggest potential avenues for novel therapeutic strategies.

## Pathogenic mechanisms of *Fusobacterium* species in disease progression

### Impact of epigenetic changes

*Fusobacterium* was once regarded as a passive resident in the gastrointestinal tract; however, it is now recognized that *F. nucleatum* infection can trigger specific tumor-associated molecular events in colorectal cancer, including the CpG island methylation phenotype, microsatellite instability, and mutations in the BRAF and TP53 genes^100,133,134^.

Epigenetic studies have shown that *F. nucleatum* can alter host cell DNA methylation and histone modification via various pathways. In particular, elevated CpG island methylation is associated with silencing of tumor suppressor genes. *F. nucleatum* has been linked to increased CpG island methylation, often mediated by DNA methyltransferases (DNMTs), enzymes that add methyl groups to DNA, thereby regulating gene expression^2,100,133,135^. This effect may be enhanced by *F. nucleatum’*s inhibition of molecules that compete with DNMTs, leading to amplified DNMT activity, further silencing tumor suppressor genes and promoting cancer progression.

Recent studies have also associated *F. nucleatum* enrichment in head and neck squamous cell carcinoma (HNSCC) with hypermethylation of tumor suppressor gene promoters. In addition, *F. nucleatum* can influence chromatin structure and regulate gene expression by modifying histones, resulting in transcriptional repression of key tumor suppressor genes^23,136,137^.

The epigenetic alterations induced by *F. nucleatum* extend to non-coding RNAs, particularly microRNAs. Several studies have shown that *F. nucleatum* can modulate host cell behavior by altering microRNA expression^138,139^, leading to enhanced inflammatory responses, increased cell proliferation, and reduced apoptosis. These intricate regulatory networks reveal how *F. nucleatum* reshapes the host epigenetic landscape, directly driving tumorigenesis and progression.

In conclusion, *F. nucleatum* plays a multifaceted role in colorectal cancer by inducing epigenetic changes. These alterations not only deepen our understanding of its contribution to cancer progression but also suggest potential therapeutic targets among these epigenetic markers. As research continues to elucidate *F. nucleatum*’s influence on the epigenetic landscape, new opportunities may arise for its integration into cancer prevention and treatment strategies.

### Activation of cell proliferation

At its core, cancer is characterized by uncontrolled cell growth, and *F. nucleatum* influences cancer cell proliferation through interactions with host proteins. The FadA–E-cadherin–β-catenin pathway is also implicated in colorectal cancer proliferation^140,141^. In colorectal cancer, *F. nucleatum* enhances tumor cell proliferation in mouse xenografts by activating Toll-like receptor 4 (TLR4) signaling via MYD88, which subsequently triggers NF-κB activation and upregulates miR-21. This microRNA downregulates RASA1, a RAS GTPase that normally restrains cell proliferation and differentiation.

In addition, *F. nucleatum* promotes intestinal tumor growth by increasing cyclin D1 expression, a key cell cycle regulator^73^. In OSCC, both *F. nucleatum* and *P. gingivalis* significantly stimulate cell proliferation through upregulation of cyclin D1 and c-Myc^142^. Activation of TLR4 by *F. nucleatum* elevates IL-6 production, which in turn activates STAT3, a transcription factor that regulates cyclin D1 and c-Myc^41^. Moreover, *F. nucleatum* induces DNA damage and promotes proliferation in oral cancer cells by reducing the expression of p27, a cyclin-dependent kinase inhibitor, and by downregulating DNA repair proteins such as Ku70 and p53 ^56^. These molecular interactions facilitate cancer cell proliferation, accelerate cell cycle progression, and impair DNA repair, contributing to tumor initiation and progression in multiple cancer types.

### Induction of inflammation

The pro-inflammatory potential of *F. nucleatum* is well-documented, as it can stimulate the production of reactive oxygen species (ROS) and pro-inflammatory cytokines^73^. Chronic inflammation is a major driver of carcinogenesis, which may partly explain the strong association between periodontitis and an increased risk of OSCC^143^. Elevated *F. nucleatum* levels correlate with increased inflammatory cytokines in both colorectal cancer and OSCC^142,144^, fostering a tumor-promoting inflammatory microenvironment.

As mentioned before, *F. nucleatum* activates the TLR4-NF-κB signaling axis, leading to pro-inflammatory cytokine production in both oral and colorectal tissues^145,146^. This inflammatory cascade reinforces tumor-supportive conditions, underscoring *F. nucleatum*’s central role in cancer progression.

### Anti-tumor immune response and immunotherapy

**Fig. 1:**
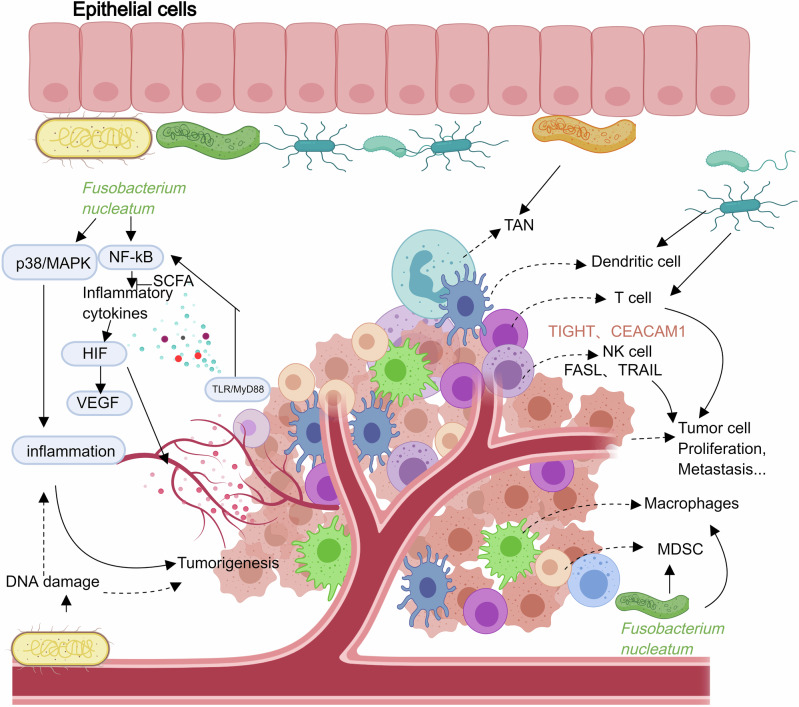
The role of *Fusobacterium* within the tumor microenvironment. The schematic depicts epithelial cells in the upper layer, with blood vessels and tumor cells in the lower portion. *F. nucleatum* activates multiple signaling pathways that stimulate the production of inflammatory cytokines, leading to inflammation and DNA damage, thereby fostering tumor initiation, proliferation, recurrence, and metastasis. In addition, *F. nucleatum* modulates the activity of various immune cells, including tumor-associated macrophages (TAMs), tumor-associated neutrophils (TANs), dendritic cells, T cells, natural killer (NK) cells, and myeloid-derived suppressor cells (MDSCs), ultimately influencing tumor growth, invasion, and dissemination. Solid lines indicate direct effects, whereas dashed lines represent indirect effects. This figure was created using *MedPeer* (medpeer.cn), and appropriate publication and licensing rights have been obtained.

As illustrated in Fig. 1, F*. nucleatum* exerts profound effects on host immune cells. It impairs dendritic cell antigen-presenting capacity, thereby weakening anti-tumor immunity, and polarizes tumor-associated neutrophils (TANs) toward a pro-tumorigenic phenotype that supports cancer cell proliferation, invasion, and metastasis. While the bulk of evidence underscores *Fusobacterium*’s immunosuppressive and tumor-promoting actions, emerging studies suggest a more nuanced role—one that may also involve modulating anti-tumor immunity and shaping responses to immunotherapy in specific contexts^147,148^. Together with its effects on dendritic cells and TANs, *F. nucleatum* also shapes other immune compartments, including myeloid-derived suppressor cells, T cells, and NK cells, thereby reinforcing an immunosuppressive tumor microenvironment.

In the ApcMin/+ mouse model, *F. nucleatum* has been shown to recruit bone marrow-derived suppressor cells, particularly myeloid-derived suppressor cells (MDSCs), to the tumor site. These MDSCs inhibit T cell proliferation and induce T cell apoptosis, fostering an immunosuppressive microenvironment^101,144^. This is supported by observations of a negative correlation between *F. nucleatum* abundance and CD3 and CD4 T cell infiltration in colorectal and breast cancer tissues. Additionally, a similar negative association exists between *F. nucleatum* load and markers of immune cells, such as B lymphocytes, CD4 T helper cells, M2 macrophages, and fibroblasts, in OSCC, underscoring *F. nucleatum*’s broad immunosuppressive influence^149^. *F. nucleatum* uses specific inhibitory proteins to impair T cell activation, arresting cells in the G1 phase of the cell cycle. The interaction between the *Fusobacterium* Fap2 adhesin and the TIGIT receptor on T cells and NK cells exemplifies an immune evasion strategy that protects both *F. nucleatum* and nearby tumor cells from immune-mediated destruction^98,150^. In addition to Fap2, other adhesins such as FadA and RadD play critical roles in *F. nucleatum*’s ability to adhere to and co-aggregate with host cells and other microbes, contributing to its pathogenic potential. Furthermore, the outer membrane proteins Fap2 and RadD induce lymphocyte cell death, and the polarization of M2 macrophages via the TLR4/IL-6/p-STAT3/c-MYC pathway reflects *Fusobacterium*’s multifaceted approach to immune suppression^151,152^.

In the context of tumor immunotherapy, *F. nucleatum* may modulate treatment effectiveness. Differences in colorectal cancer microsatellite instability (MSI) status show that high levels of *F. nucleatum* correlate with an enhanced response to PD-1 blockade therapy^100,153^. In mouse models of colorectal cancer, *F. nucleatum* not only amplifies the effects of PD-L1 inhibitors but also synergizes with these therapies to extend survival. As has been observed in OSCC, *F. nucleatum* can induce PD-L1 expression, potentially via the STING signaling pathway, which may contribute to resistance or response modulation in PD-L1 blockade therapy^154–156^. Furthermore, patient-derived organoid models have shown a positive association between *F. nucleatum* levels and response to PD-L1 blockade, highlighting the bacterium’s potential as a biomarker or adjunct in colorectal cancer immunotherapy. Beyond single-taxon effects such as *F. nucleatum*, system-level modulation of the gut ecosystem via fecal microbiota transplantation (FMT) can reshape responses to immune checkpoint inhibitors (ICIs). However, evidence remains mixed and context-dependent, underscoring the need for standardized, patient-tailored FMT–ICI protocols and rigorous multicenter randomized trials^157^. In parallel, analogous microbiota-transfer strategies along the oral–gut axis, such as transplanting defined oral microbial consortia to recalibrate dysbiosis, may likewise be explored to achieve disease-modifying benefits.

### Cell migration and invasion

Matrix metalloproteinases (MMPs) are zinc-dependent endopeptidases responsible for degrading extracellular matrix (ECM) components, and play a key role in pathological processes that involve excessive ECM degradation, such as tumor invasion and metastasis. Both *P. gingivalis* and *F. nucleatum* can stimulate MMP production through distinct mechanisms, promoting cancer cell invasion and metastasis^158,159^.

In OSCC, periodontal pathogens like *P. gingivalis* and *F. nucleatum* have been shown to induce the expression of MMPs, particularly MMP-1 and MMP-9^160^. In breast cancer models, co-culture of AT3 mouse breast carcinoma cells with *F. nucleatum* resulted in overexpression of MMP-9, implicating this bacterium in enhancing metastatic potential^161,162^.

*F. nucleatum* is also associated with the dysregulation of genes involved in the EMT, a process critical for cell migration and invasion. In colorectal cancer, elevated *F. nucleatum* levels correlate with decreased expression of the epithelial marker E-cadherin and increased expression of the mesenchymal marker N-cadherin^163^. Similarly, OSCC cell lines exposed to *F. nucleatum* exhibit downregulation of E-cadherin and upregulation of N-cadherin, vimentin, and Snail^142^. These changes signify an EMT-like process, essential for cancer cells to acquire invasive properties.

*F. nucleatum* has been shown to upregulate ZEB1, promoting a mesenchymal phenotype in oral cancer cells^142^. This mechanism mirrors bacterial modulation of EMT observed in *Helicobacter pylori*-infected gastric epithelial cells, suggesting a conserved role for bacterial influence on EMT in cancer progression.

### Co-aggregation and oral carcinogenesis

*F. nucleatum* acts as a keystone species in both the oral and gastrointestinal microbiomes, often serving as a bridge for co-aggregation among various microbial species. This ability to facilitate multispecies biofilm formation largely stems from its outer membrane adhesin, Fap2, which mediates interactions with other bacteria, including both Gram-negative and Gram-positive species^164^.

In the oral cavity, *F. nucleatum* can significantly alter the local environment, affecting the colonization and pathogenic potential of other microbes. For example, in the presence of *P. gingivalis*, a key periodontal pathogen, *F. nucleatum* is consistently observed, suggesting that its colonization precedes, and is necessary for, that of *P. gingivalis*. Through the fermentation of glutamate and aspartate, *F. nucleatum* produces ammonia, which neutralizes acidic metabolic byproducts like lactic acid, creating a more favorable environment for *P. gingivalis* and promoting a synergistic relationship within the biofilm^165^.

The co-aggregation of *F. nucleatum* with *P. gingivalis* has been implicated in both periodontal disease progression and tumor development. *F. nucleatum* facilitates multispecies assemblies through outer membrane adhesins such as Fap2 and RadD. It promotes the colonization of *P. gingivalis* and *C. albicans* by neutralizing local acidity via ammonia production, thereby creating a favorable environment for microbial synergy. Together, they may contribute to tumorigenesis by inducing chronic inflammation and stimulating OSCC cell proliferation in vitro^142^. Additionally, *F. nucleatum* modulates the immune response, potentially suppressing host defenses and facilitating the persistence of other pathogens.

Interactions between *Fusobacterium* species and *Candida albicans* (*C. albicans*), an opportunistic pathogenic yeast found in the gastrointestinal tract and oral cavity, further illustrate the role of this co-aggregation in disease progression. One proposed mechanism involves *Candida*-derived ethanol dehydrogenase converting ethanol into acetaldehyde, a recognized carcinogen in the oral environment. Co-aggregation between *Fusobacterium* species and *Candida* enhances colonization^166^, and elevated levels of *F. nucleatum* have been observed in *Candida*-colonized oral leukoplakia lesions. This co-localization may increase epithelial exposure to acetaldehyde and contribute to early-stage malignant transformation, although this remains to be clinically validated. Further studies are warranted to confirm the role of *Fusobacterium*-*Candida* synergy in the pathogenesis of OSCC.

In conclusion, the complex network of interactions between *Fusobacterium* and other microbes within the oral and gastrointestinal microbiomes plays a pivotal role in shaping its impact on health and disease. These interactions drive chronic inflammation, modulate immune responses, and contribute to genotoxic stress, collectively promoting carcinogenesis and other pathological conditions^167^.

## Association with other related diseases and cancers

**Fig. 2:**
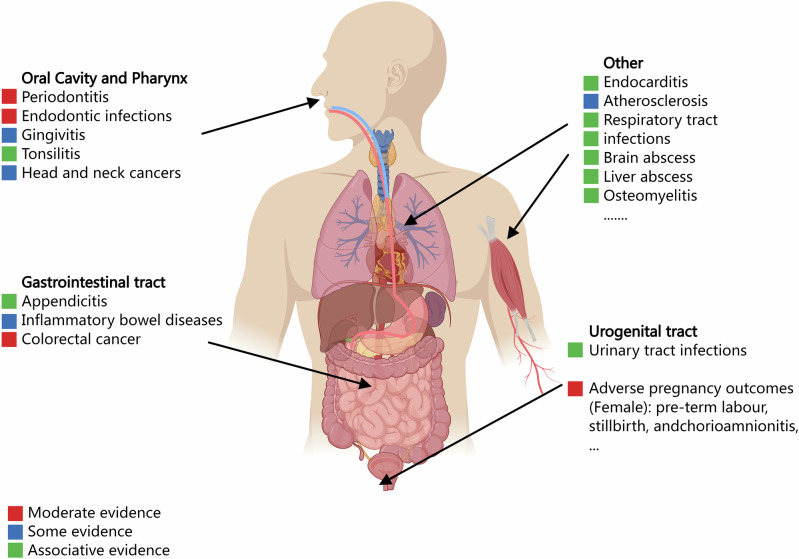
Association between microbiota and systemic diseases. The left side highlights major diseases linked to microbiota in the oral cavity and pharynx, including periodontitis, endodontic infections, gingivitis, tonsillitis, and head and neck cancers. The central human figure indicates bodily systems potentially impacted by microbiota-related conditions, including the gastrointestinal tract, respiratory system, and urogenital tract. In the gastrointestinal tract, specific conditions such as appendicitis, inflammatory bowel diseases, and colorectal cancer are shown. The right side lists additional diseases associated with microbiota, such as endocarditis, atherosclerosis, respiratory tract infections, brain abscess, liver abscess, and osteomyelitis. In females, microbiota imbalances may also be linked to adverse pregnancy outcomes, including preterm labor, stillbirth, and chorioamnionitis. The color coding represents the strength of evidence linking these diseases to microbiota: red for moderate evidence, orange for some evidence, and green for associative evidence. This figure was created using *MedPeer* (medpeer.cn), and appropriate publication and licensing rights have been obtained.

*Fusobacterium*-related diseases span a range of conditions, reflecting the bacterium’s broad impact on human health (Fig. 2). Since its initial association with infections in 1936, particularly jugular vein septic thrombophlebitis, *Fusobacterium* has been increasingly implicated in various health conditions. The rising incidence of *Fusobacterium* infections is likely due to improved detection methods and changes in antibiotic use^168^. These infections, especially severe in pediatric cases, frequently affect the head and neck, with acute otitis media being a prominent manifestation^169–173^. Rapid progression of these infections can lead to serious complications, including bacteremia and osteomyelitis^174^, underscoring the need for early detection and treatment.

Lemierre’s syndrome, a severe form of septic thrombophlebitis affecting the internal jugular vein, exemplifies the significant morbidity associated with *Fusobacterium* infections, particularly those involving *F. necrophorum*^175–177^. The bacterium’s role in systemic infections, such as bacteremia in individuals with malignancies or immunosuppression, highlights its potential for high mortality rates and necessitates vigilant monitoring and comprehensive management^178^. *Fusobacterium*-related endocarditis, though rare, poses a serious risk to individuals with preexisting heart conditions^179^.

Recent large-scale analyses of the global burden and inequalities associated with COVID-19 underscore its far-reaching health impacts beyond acute infection, raising questions about potential effects on the human microbiome^180,181^. Indeed, emerging evidence suggests that the COVID-19 pandemic may influence the oral and gut microbiome, with some research reporting shifts in *Fusobacterium* abundance; however, the clinical relevance of this observation remains to be clarified^182^. Some preliminary studies have reported increased *Fusobacterium* levels in individuals with gout, but whether this reflects a causal relationship or secondary microbial shift remains unclear^183,184^. This expanding association between *Fusobacterium* and various health conditions reinforces the need for advanced research and targeted therapeutic strategies. However, these associations remain speculative and should be interpreted with caution. Further longitudinal and mechanistic studies are needed to validate any potential causative links.

Beyond its well-documented connection to colorectal and oral cancers, *Fusobacterium* has been implicated in other malignancies, including breast and pancreatic cancers. Studies have detected *Fusobacterium* in breast cancer tissues, suggesting a potential role in cancer progression, and have associated its presence with poorer prognostic outcomes^101,185–187^. *F. nucleatum* has also been detected in liver abscesses and in advanced liver cancer, suggesting it may contribute to liver cancer progression and could serve as a therapeutic target^188^.

The association between *Fusobacterium* and diverse cancer types has become a focal point of research, with recent studies linking *F. nucleatum* to breast cancer through Fap2-mediated adhesion to elevated levels of the Gal-GalNAc antigen in breast tissues^161^. *F. nucleatum*-derived extracellular vesicles stimulate breast cancer proliferation and metastasis, potentially through TLR4 activation^186^. Moreover, *F. nucleatum* may promote breast cancer cell proliferation and treatment resistance by stimulating autophagy and facilitating immune evasion through immune suppression^189^. In liver cancer, *F. nucleatum* has been detected in liver abscesses and in patients with advanced disease^190^. Although the exact mechanisms remain unclear, they may involve pathways similar to those observed in breast cancer, including enhanced tumor growth and metastasis.

Research into *Fusobacterium*’s role in cancer also examines its interactions within the hypoxic tumor microenvironment^191^. Hypoxia, common in solid tumors, affects tumor cell behavior and their response to bacterial infections^191,192^. Understanding how *F. nucleatum* interacts with hypoxic tumor cells could uncover new insights into its role in cancer progression and suggest potential therapeutic approaches.

In summary, the growing body of research on *Fusobacterium* and its involvement in cancers beyond the oral and gastrointestinal tracts underscores its potential as a therapeutic target. Novel therapies, such as *F. nucleatum*-mimicking nanovehicles, which utilize *F. nucleatum*’s surface characteristics to target specific cancer cells, show promise in improving the treatment of cancers^193,194^. These nanovehicles can be engineered to deliver therapeutic agents directly to *Fusobacterium*-enriched tumor sites, enhancing the specificity and efficacy of cancer treatments. As research in this area advances, the intricate relationship between *Fusobacterium* and cancer is likely to reveal additional intervention opportunities.

In conclusion, expanding research on *Fusobacterium*, particularly *F. nucleatum*, underscores its multifaceted role beyond the oral cavity, linking it to a wide spectrum of systemic diseases, from inflammatory bowel disease to colorectal, pancreatic, and esophageal cancers. Acting both as a commensal organism and a potent pathogen, *Fusobacterium* serves as a microbial bridge within complex communities, facilitating co-aggregation, shaping ecosystem dynamics, and influencing disease pathogenesis through inflammation, cell proliferation, immune modulation, and tumor microenvironment remodeling. Its detection in malignancies beyond the colorectum suggests a broader oncogenic potential that warrants deeper investigation into its molecular mechanisms and host interactions. Clinically, its involvement in systemic infections such as Lemierre’s syndrome, bacteremia, and cervicofacial and gastrointestinal infections further highlights its significance. As the understanding of *Fusobacterium*-driven tumorigenesis advances, emerging therapeutic strategies, such as nanovehicle-based targeted drug delivery and novel antibiotics directed at specific strains, offer promising avenues to enhance treatment efficacy, minimize side effects, and improve patient outcomes. These innovations, coupled with continued elucidation of *Fusobacterium*’s pathogenic mechanisms, could transform both the prevention and management of its associated diseases.

## Data Availability

No datasets were generated or analysed during the current study.
